# Bioactive Molecules Release and Cellular Responses of Alginate-Tricalcium Phosphate Particles Hybrid Gel

**DOI:** 10.3390/nano7110389

**Published:** 2017-11-14

**Authors:** Dipankar Das, Sumi Bang, Shengmin Zhang, Insup Noh

**Affiliations:** 1 Convergence Institute of Biomedical Engineering and Biomaterials, Seoul National University of Science of Technology, 232 Gongneung-ro, Nowon-gu, Seoul 01811, Korea; dipankardas@seoultech.ac.kr (D.D.); bobosumi48@gmail.com (S.B.); 2 Department of Chemical and Biomolecular Engineering, Seoul National University of Science of Technology, 232 Gongneung-ro, Nowon-gu, Seoul 01811, Korea; 3 Advanced Biomaterials and Tissue Engineering Center, Huazhong University of Science and Technology, Wuhan 430074, China; smzhang@mail.hust.edu.cn

**Keywords:** alginate, α-TCP particles, drug delivery, hybrid gel, MC3T3 cell responses

## Abstract

In this article, a hybrid gel has been developed using sodium alginate (Alg) and α-tricalcium phosphate (α-TCP) particles through ionic crosslinking process for the application in bone tissue engineering. The effects of pH and composition of the gel on osteoblast cells (MC3T3) response and bioactive molecules release have been evaluated. At first, a slurry of Alg and α-TCP has been prepared using an ultrasonicator for the homogeneous distribution of α-TCP particles in the Alg network and to achieve adequate interfacial interaction between them. After that, CaCl2 solution has been added to the slurry so that ionic crosslinked gel (Alg-α-TCP) is formed. The developed hybrid gel has been physico-chemically characterized using Fourier transform infrared (FTIR) spectroscopy, scanning electron microscopy (SEM) and a swelling study. The SEM analysis depicted the presence of α-TCP micro-particles on the surface of the hybrid gel, while cross-section images signified that the α-TCP particles are fully embedded in the porous gel network. Different % swelling ratio at pH 4, 7 and 7.4 confirmed the pH responsiveness of the Alg-α-TCP gel. The hybrid gel having lower % α-TCP particles showed higher % swelling at pH 7.4. The hybrid gel demonstrated a faster release rate of bovine serum albumin (BSA), tetracycline (TCN) and dimethyloxalylglycine (DMOG) at pH 7.4 and for the grade having lower % α-TCP particles. The MC3T3 cells are viable inside the hybrid gel, while the rate of cell proliferation is higher at pH 7.4 compared to pH 7. The in vitro cytotoxicity analysis using thiazolyl blue tetrazolium bromide (MTT), bromodeoxyuridine (BrdU) and neutral red assays ascertained that the hybrid gel is non-toxic for MC3T3 cells. The experimental results implied that the non-toxic and biocompatible Alg-α-TCP hybrid gel could be used as scaffold in bone tissue engineering.

## 1. Introduction

Hydrogels are hydrophilic, three-dimensional (3-D), physically or chemically crosslinked polymer networks which absorb large amount of water in aqueous medium or biological fluids [1,2]. The excellent swelling nature of the hydrogel helps to retain water molecules into the 3-D network that aids in keeping biocompatibility, structural integrity and elasticity [1]. Owing to these features, hydrogels have been extensively employed in different biomedical applications such as drug delivery [1,2,3], protein delivery [3], tissue engineering [4,5,6,7] and regenerative medicine [7]. Researchers have also explored that the incorporation of micro/nano-sized fillers into polymeric hydrogels forms composite hydrogels with improved mechanical properties, unique rheological behaviors, promising degradation features and enriched bioactivities [8,9,10]. However, the direct incorporation of inorganic fillers leads to unstable mixtures of the polymeric and inorganic phases [10]. This causes isolation of the inorganic particles from the hydrogel network over a prolonged period of time, or during the swelling-deswelling process, because of the absence of strong interactions between the inorganic particles and the polymer matrix [10]. Recently, hybrid hydrogels composed of both flexible polymer and stiff inorganic components are growing as interdisciplinary research materials that offer striking candidates for the design of injectable composite material towards bone tissue engineering. Indeed, the demands of bone regeneration or repair are increasing since population and average lifetime of human beings continue escalating. The incorporation of an inorganic constituent into the polymeric hydrogel matrix may afford ample mechanical strength, pioneer osteoconductive or osteoinductive properties and provides nucleation sites for in vivo bone formation [11,12]. The injectable hybrid hydrogels are being used in 3-D printing technology for tissue engineering with specific architectonics and properties because of the printing ability of the gels [13]. While the efficacy of 3-D bioprinting materials depends on the successful design of the in situ encapsulation of live cells into, and delivery of, bioactive molecules from materials [13]. However, for ideal tissue engineering, hydrogel scaffolds should contain enough porosity, interconnection channels for transport nutrients, biocompatibility, biodegradability and high mechanical strength [14,15]. Porous degradable scaffolds give a precise atmosphere for cells/tissue growth in vivo and vitro [14]. High porosity of scaffold facilitates attachment and proliferation of bone cells [16,17]. Macro size pores (pores > 100 µm) are recognized to contribute to osteogenesis by assisting cell and ion transport [18]. Micro size pores (pores < 20 µm) are considered to increase bone growth into scaffolds by affording connection positions for osteoblasts and mounting surface area for protein adsorption [19]. Additionally, high interconnectivities between pores are plenty for regular cell distribution when seeded [20].

Among the materials developed and employed for tissue engineering scaffolds, synthetic biodegradable polymers are imperative class of materials as their properties can eagerly be adapted by the control of the polymerization reaction [14,21]. But, the major disadvantage of the synthetic biodegradable polymer scaffolds is lack of the bioactivity [14]. In this aspect, hydrogel scaffolds fabricated from natural polymers have potential advantages—for instance biodegradability, biocompatibility, antithrombotic and hemostatic properties, incredible healing activity, water retention capacity, antibacterial activity, immunological property and are low-cost [14,22]. Among natural polymers, alginic acid (alginate, Alg) has been extensively used in the synthesis of hydrogels for biomedical applications owing to its biocompatibility, biodegradability and inexpensiveness [23]. Alg is an anionic polysaccharide composed of d-mannuronic acid (M block) and l-guluronic acid (G block) [23]. Mannuronic acid forms β-1,4-glycosidic linkages, while guluronic acid forms α-1,4-glycosidic bonds. Alg has ability to form gels upon addition of multivalent ions [23]. Moreover, Alg is mucoadhesive, biocompatible and non-immunogenic making it very suitable for tissue engineering applications [23]. The Alg-based hydrogels have been comprehensively applied in drug/gene delivery, tissue engineering, wound healing, cell encapsulation and so on [24,25]. Alg hydrogel as scaffold for tissue engineering has distinctive advantages, for instance, they can be mixed with the cells in liquid form into the body to fill the damaged tissue and availability of three-dimensional space for cells growth [23]. Because of the high rate of degradation in cell culture and the weak mechanical properties, a single component Alg hydrogel restricts the practical application [23]. To overcome these issues, Alg has been modified—either physically or covalently—with a range of components such as gelatin, collagen, calcium phosphate, chitin whiskers, silica, grapheme oxide and bioglass to achieve hybrid materials with sufficient mechanical strength for drug delivery and different biological applications [13,23,24,25,26,27,28,29,30,31,32,33,34,35,36]. In recent years, bioceramics such as hydroxyapatite (HAp) and α-tricalcium phosphate (α-TCP) have often been employed with natural polymers for bone tissue regeneration because of the chemical resemblance of the materials to the mineral phase of bone [37,38]. Among the various bone prosthetic materials, TCP endures time-dependent absorption in vivo and is thus superior to HAp and other materials with low solubility in vivo regarding replacement by bone [39]. Moreover, the addition of α-TCP not only amplifies the mechanical properties but also increases the osteoconductivity of the fabricated scaffolds, which is complimentary for bone tissue engineering [16,40].

Here, we report the development of a hybrid hydrogel of sodium alginate (Alg) and alpha tricalcium phosphate (α-TCP) particles. Three different compositions (10:1, 2:1 and 1:10 *w*/*w*) have been prepared using α-TCP at varying quantities in 6% Alg solutions using an ultrasonicator. To achieve regular distributions of bioceramics (α-TCP) and for better interfacial interaction between Alg and α-TCP, ultrasonicator has been applied. A self-hardening of scaffold is formed by crosslinking of Alg in presence of calcium chloride (CaCl_2_) solution. The effects of pH and composition towards cellular responses and bioactive molecules release from ionically crosslinked hybrid gel (Alg-α-TCP) have also been studied. The bovine serum albumin (BSA) as the model protein, tetracycline (TCN) as the model antibiotic, and dimethyloxalylglycine (DMOG) as the model angiogenic/osteogenic drug were in-situ incorporated into the hybrid gels and the in vitro release nature has been studied at pH 4, 7 and 7.4 at 37 °C. BSA has 76% sequence uniqueness with human serum albumin [41,42]. It is involved in various physiological activities like acid-base balance, transport and binding of numerous types of drugs and delivery of fatty acids [41,42,43,44]. DMOG is used to improve wound healing, attenuate post-ischemic myocardial injury and renal injury in remnant kidney [45]. It can increase angiogenic activity of bone marrow stromal cells and adipose derived stem cells via promoting the expression of hypoxia inducible factor—1α (HIF-1α) and its downstream genes in cells to improve the angiogenesis of tissue-engineered bone [46]. While TCN shows anti-collagenase activity, inhibition of bone resorption, anti-inflammatory action and having capability to promote attachment of fibroblasts and connective tissue to root surfaces [47,48]. Finally, the ionic crosslinked Alg-α-TCP hybrid gel, which is non-toxic and biocompatible against MC3T3 cells and executed controlled BSA, TCN and DMOG release ability, could be used as scaffold in bone tissue engineering.

## 2. Materials and Methods

### 2.1. Materials

Alginic acid (Alg) sodium salt from brown algae (medium viscosity), calcium chloride, calcium hydrogen phosphate dihydrate, calcium carbonate (purity ≥ 99%), bovine serum albumin (BSA, molecular weight (MW) ~66 kDa, lyophilized powder) and tetracycline (TCN, MW 444.43 Da, anhydrous basis) were purchased from Sigma Aldrich (St. Louis, MO, USA). Dimethyloxalylglycine (DMOG, MW 175.14 Da, purity ≥ 98%) was purchased from Cayman Chemical Company (Ann Arbor, MI, USA). Double distilled water (DW) was employed for experimental purposes.

### 2.2. Synthesis

#### 2.2.1. Synthesis of α-TCP by Ball Milling Machine

The α-TCP was prepared by the same protocol as described in our previous report [49]. Briefly, 100 mL ethanol was added to a molar mixer (2:1) of calcium hydrogen phosphate dihydrate (68.84 g) and calcium carbonate (20.02 g). The mixture was placed into a 500 mL alumina pot containing 100 g of zirconia particles (*d* = 5 mm). It was then mixed in the ball milling machine (Model: SBML-2, SciLab Korea Co., Ltd., Seoul, Korea) at 150 rpm for 24 h. The zirconia particles were then recovered using a sieve (Daehan Scientific, Seoul, Korea) with 425 µm pores. The dicalcium phosphate and calcium carbonate powders were then transferred to an aluminum tray and kept in a heating oven at 70 °C for 24 h for the removal of ethanol [49]. Afterwards, the powder containing alumina pot was heated up in an electrical furnace (Model: MHS-160526-01, MiR furnace, Seoul, Korea) at 1300 °C for 16 h using the provided program (escalation of heating for 2 h and heating for 16 h and then cooling). After cooling, clusters of α-TCP particles were acquired. The α-TCP clumps were transferred into an engineering plastic pot containing mixture of two kinds of zirconia particles (300 g with *d* = 20 mm and 100 g with *d* = 4 mm) and 300 mL of ethanol. The plastic pot was loaded in the ball milling machine and rotated at room temperature for 24 h [49]. Finally, the dried α-TCP particles were collected after evaporating of ethanol at 70 °C in a heating oven. The particle size of α-TCP particle was measured by laser diffraction particle size analyzer (Model: LS I3 320, Beckman Coulter, Brea, CA, USA) and employed for hybrid gel formation.

#### 2.2.2. Preparation of Sodium Alginate (Alg) and α-TCP Particles Hybrid Gel

**Figure 1:**
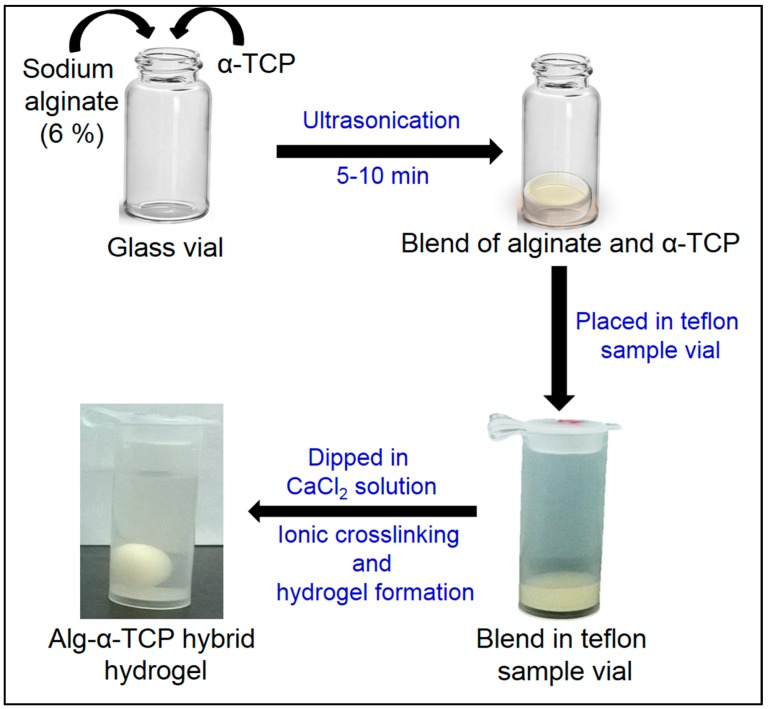
Schematic presentation of the fabrication process of Alg-α-TCP hybrid gel.

The hybrid gel of Alg and α-TCP particles was fabricated by ionic crosslinking as follows: Firstly, sodium alginate (0.3 g) and α-TCP particles (0.03, 0.15 and 3.0 g) were poured into 5 mL distilled water (pH 7.0) in a 25 mL glass vial (Figure 1). After that, the components were mixed using the ultrasonicator (Vibra Cell, Sonics, Gyeonggi-do, Korea) to get a homogeneous slurry [49]. Then, a definite volume of blend (0.3 mL) was kept in an 8 mL Teflon sample vial (*d* = 10.3 mm, Daihan Scientific, Seoul, Korea) and 0.1 M calcium chloride (CaCl_2_) solution was poured into the vial for ionic crosslinking. After that, the round shape gel was formed and taken out from calcium chloride solution. The gel samples were washed three times with distilled water (3 × 50 mL) to remove excess calcium ions present in the surface of the gel. Finally, the gel mass was dried in a lyophilizer (IL Shin Lab, Gyeonggi-do, Korea) for 48 h at −60 °C and used for further study.

### 2.3. Determination of Ionic Concentration Dependent Gelation Time of Hybrid Gel

The gelation times of the Alg-α-TCP mixtures (10:1, 2:1 and 1:10 in *w*/*w*) were measured by taking 0.3 mL slurry in the 8 mL Teflon sample vials (*d* = 10.3 mm, Daihan Scientific, Seoul, Korea). To observe the effect of ionic concentration of CaCl_2_ on gelation time, different concentrations of calcium chloride solutions (0.05, 0.1, 0.2, 0.5 and 1.0 M) were poured in the vials at room temperature (25 °C). Gelation phenomenon was visually observed when the slurry converted into round shape and detached from the bottom of the vials.

### 2.4. Characterizations

The attenuated total reflectance Fourier transform infrared (ATR-FTIR) spectra of sodium alginate, α-TCP and dried hybrid gels (10:1, 2:1 and 1:10 in *w*/*w*) were recorded using an ATR-FTIR spectrometer (Model: Travel IR, Smiths Detection, Edgewood, WI, USA). The wavelength range of spectra were 650–4000 cm^−1^. The surface morphology of α-TCP and the dried hybrid gels (10:1, 2:1 and 1:10) were observed using scanning electron microscopy (Model: SEM, TESCAN VEGA3, Tescan, Seoul, Korea). The particle size of α-TCP was determined using a laser diffraction particle size analyzer (Model: LS I3 320, Beckman Coulter, Brea, CA, USA) at solid state. The powder X-ray diffraction (XRD) analysis of α-TCP was carried out using X-ray diffractometer (Model: Bruker DE/D8 Advance, Bruker AXS GmbH, Karlsruhe, Germany). The step size was 0.02°, time per step was 0.1 s. The range of 2θ was 5°–80°. The calcium content of the Alg-α-TCP (10:1, 2:1 and 1:10) hybrid gels was measured by inductively coupled plasma mass spectrometry (ICP-MS) (Model: iCAP Q, ThermoFisher Scientific, Bremen, Germany).

### 2.5. Determination of Calcium Content in Alg-α-TCP Hybrid Gels

The calcium (Ca) content in the Alg-α-TCP (10:1, 2:1 and 1:10) hybrid gels were measured by inductively coupled plasma mass spectrometry (ICP-MS) (Model: iCAP Q, ThermoFisher Scientific, Bremen, Germany). A known amount (0.05 g) of each dried gel was treated with 70% nitric acid (HNO_3_) solution for 2 days at 110 °C for the formation of water soluble calcium nitrate compound. After that, the solutions were filtered. The filtrates were transferred in different 50 mL volumetric flasks and the volume was make up by distilled water. The calcium content in each solution was determined three times by inductively coupled plasma mass spectrometer (Model: iCAP Q, ThermoFisher Scientific, Bremen, Germany). The average concentration of the Ca in the solution is expressed in parts per billion (ppb, µg/L).

### 2.6. Swelling Study

The % swelling of the hybrid gels (10:1, 2:1 and 1:10 in *w*/*w*) were measured at pH 4, 7 and 7.4 and 37 °C. In brief, the pre-weighed dried hybrid gels (0.25 g) were immersed in 50 mL different buffers (pH 4, 7 and 7.4) for 12 h. After regular time interval (1 h), the swollen gels were taken out from distilled water and the surface water was blotted off by tissue paper. The hydrogels were then reweighed until constant weights were attained. The % swelling was calculated by the Equation (1):

(1) $$\mathrm{Swelling}\left(\%\right)=\frac{\mathrm{Final}\;\mathrm{weight}\;\mathrm{of}\;\mathrm{hybrid}\;\mathrm{gel}-\mathrm{Initial}\;\mathrm{weight}\;\mathrm{of}\;\mathrm{dried}\;\mathrm{hybrid}\;\mathrm{gel}}{\mathrm{Initial}\;\mathrm{weight}\;\mathrm{of}\;\mathrm{dried}\;\mathrm{hybrid}\;\mathrm{gel}}\text{×}100$$

### 2.7. MC3T3 Cell Viability inside the Alg-α-TCP Hybrid Gel

An osteoblast cell line derived from Mus musculus (mouse) calvaria (MC3T3, Sigma Aldrich Sigma Aldrich Co., St. Louis, MO, USA) was *in vitro* cultured in alpha minimum essential medium (α-MEM) containing both 10% fetal bovine serum (Gibco Korea, Seoul, Korea) and penicillin-streptomycin (100 unit/mL) in an incubator with 5% CO_2_ at 37 °C. At first, Alg-α-TCP hybrid mixtures (10:1, 2:1 and 1:10) were sterilized by autoclave (AC-02, Jeio Tech; Seoul, Korea). After wards, the MC3T3 cells were put on the surface of 0.2 mL blends at a density of 1 × 10^4^ cells/mL in 24 well plates by syringe and incubated for 5 min. After that, the cells were covered by additional 0.2 mL of blends. Next, 100 µL mixture (9:1) of α-MEM media and 0.1 M CaCl_2_ solution was added two times in each well for 20 min. Again, 100 µL mixture (9:1) of α-MEM media and 0.1 M CaCl_2_ solution was added in each well for 15 min to form hybrid gel. After removing mix solution from wells, the hybrid gels were washed 3 times with phosphate buffered saline (pH 7.4) for 3 min. Subsequently, 1.5 mL of α-MEM media was supplied to each well and changed after 24 h. The cells inside of the hybrid gels were cultured up to 10 days. To observe the effect of pH on proliferation and viability, cell study was performed at pH 7.0 and 7.4.

Assay of live and dead viability for mammalian cells was carried out following the protocol of the vendor (Invitrogen, Carlsbad, CA, USA). Both 1.2 μL of 2 mM ethidium homodimer-1 (EthD-1) and 0.3 μL of 4 mM calcein-acetoxymethyl ester (Calcein AM) were added into 600 μL phosphate buffer saline (PBS). After addition of the prepared agents to each well and 30 min of incubation, the images of the cells inside the hybrid gels were captured by fluorescence microscope (Leica DMLB, Wetzlar, Germany).

### 2.8. Cytotoxicity Study of Alg-α-TCP Hybrid Gel Using MTT, Brd U and Neutral Red Assays

The cytotoxicity study was performed with the thiazolyl blue tetrazolium bromide (MTT assay), bromodeoxyuridine (BrdU) assay and neutral red assay. The method is described in below:

#### 2.8.1. MTT Assay

After seeding 1 × 10^4^ no. of MC3T3 cells in a 96 well-plate, the samples were incubated in an incubator with 5% CO_2_ atmosphere at 37 °C for 24 h. Afterwards, medium was removed and 100 mL each extracted solutions of positive control Teflon, negative control latex (1 × 1 cm^2^) and three hybrid gels (10:1, 2:1 and 1:10) were added into each cell well and cultured for another 24 h. Next, 20 μL MTT solution (2 mg/mL in PBS) was added in the culture medium and lasted for another 4 h. Then, medium was removed and 100 μL dimethyl sulfoxide (DMSO) was poured. The optical density of the final solution was measured by microplate reader at the wavelength of 570 nm.

#### 2.8.2. BrdU Assay

The same protocol was followed for the culture of MC3T3 cells and mixing of five extracted solutions as performed above for MTT assay. After that, 10 μL BrdU labeling solution was added into the culture medium and kept in the incubator for another 2 h. Then, 200 μL Fixodent solution and 100 μL anti-BrdU peroxide-labeled anti-BrdU antibody were added to each well according to the manufacturer’s protocol. After washing the wells, 25 μL 1.0 M H_2_SO_4_ solution was added into each well. The optical density of the samples was measured by microplate reader at the wavelength of 450 nm with the reference of 690 nm.

#### 2.8.3. Neutral Red Assay

After cultured of MC3T3 cells for 24 h, the addition of five kinds of extracted solutions of Teflon, latex and three hybrid gels (10:1, 2:1 and 1:10) was performed according to same procedure as described above for MTT and BrdU assays. Subsequently, the mixture of the culture medium and 0.33% neutral red solution (9:1) were added into the well plate and the cell culture was lasted for another 2 h in the incubator. After 10 min of washing with the fixation solution, 100 μL solubilizing solution was added into each well. The optical density was measured at the wavelength of 550 nm by microplate reader (Tecan GENios FL., GMI, Brooklyn Park, MN, USA) with the reference of 690 nm.

### 2.9. In-Situ Encapsulation of Protein (BSA) and Drugs (TCN and DMOG) into Hybrid Gel

The *in situ* incorporation of bovine serum albumin (BSA), tetracycline (TCN) and dimethyloxalylglycine (DMOG) into different grades of hybrid gel were performed at room temperature (25 °C) using distilled water as media (pH 7.0) using the ultrasonicator. For this experiment, the same mole of BSA, TCN and DMOG (4.95 × 10^−6^ mol) were homogeneously mixed with different ratios (10:1, 2:1 and 1:10) of Alg/α-TCP (0.165 g) and then put into 8 mL Teflon sample vial (*d* = 10.3 mm, Daihan Scientific, Seoul, Korea). After that, 0.1 M CaCl_2_ solution was added and kept 30 min for crosslinking and round shape gel formation. After that, protein and drugs incorporated gel beads were dried in lyophilizer for 48 h and used for release study.

### 2.10. In Vitro Protein and Drugs Release Study

The in vitro release study of BSA, TCN and DMOG incorporated hybrid gels were executed in different buffers (pH 4, 7 and 7.4) at 37 °C. The BSA, TCN and DMOG release were determined spectrophotometrically using a UV-Vis spectrophotometer (BioMate 3S, ThermoScientific, Madison, WI, USA). Briefly, dried BSA, TCN and DMOG incorporated gels were kept in 10 mL distilled water into 25 mL screw Teflon bottles (Daihan Scientific, Seoul, Korea). After definite intervals, the solutions were taken out from the Teflon bottles and the spectra were recorded. After each measurement, the solutions were returned back to the bottles. On the basis of standard solutions, percentage of BSA, TCN and DMOG were calculated.

### 2.11. Statistical Analysis

All data were expressed as mean ± standard deviations. Statistical significance was assessed with one-way and multi-way ANOVA by employing the SPSS 18.0 program (ver. 18.0, SPSS Inc., Chicago, IL, USA). The comparisons between two groups were carried out, using *t*-test and the samples were considered as significantly different when *p* < 0.05.

## 3. Results and Discussion

### 3.1. Synthesis of α–TCP Particles

Wet milling process was applied for the synthesis of α-TCP particles using solid precursors (calcium hydrogen phosphate dihydrate and CaCO_3_) in ethanol at 1300 °C. The precursors were milled concurrently to make small particles by improving the contact space [50]. At 1300 °C, dicalcium hydrogen phosphate reacted with calcium carbonate and formed TCP as describe in Equation (2) [50].

(2) $$2[{\mathrm{CaHPO}}_{4}\cdot 2H_{2}O]+{\mathrm{CaCO}}_{3}={\mathrm{Ca}}_{3}{\left({\mathrm{PO}}_{4}\right)}_{2}+{\mathrm{CO}}_{2}+5H_{2}O$$

**Figure 2:**
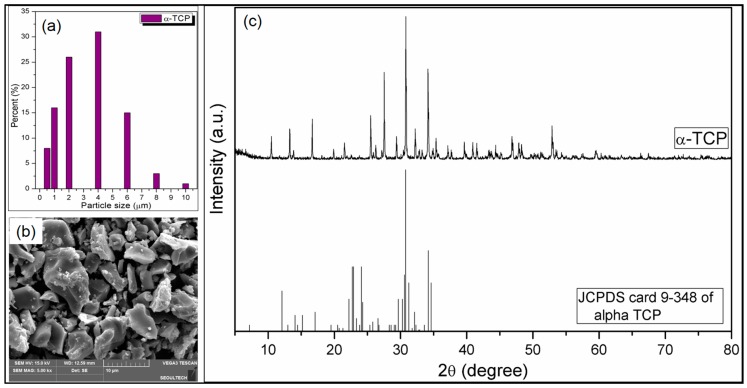
(**a**) Size distributions of the synthesized α-TCP particles obtained from laser diffraction micro-particle size analyzer; (**b**) Scanning electron microscopy (SEM) image of the synthesized α-TCP particles; (**c**) X-ray diffraction (XRD) spectra of the synthesized α-TCP particles and JCPDS card 9-348 of alpha TCP.

The size of synthesized α-TCP particles has been determined by laser diffraction micro-particle size analyzer. The result is depicted in Figure 2a. It is obvious that the particle sizes of the α-TCP remain between 0.5 and 10 µm. The SEM image of the α-TCP particles (Figure 2b) suggests that different shaped α-TCP particles formed, while particle sizes remain within micrometer range. Figure 2c depicts the XRD spectra of the synthesized α-TCP particles and standard JCPDS card 9-348 of alpha TCP [51]. From Figure 2c, it is observed that the major diffraction peak of α-TCP appeared at 2θ = 31°, which is well matched with the major diffraction peak of JCPDS card 9-348 of alpha TCP, confirming the formation of α-TCP.

### 3.2. Fabrication of Alg-α-TCP Hybrid Gel

**Figure 3:**
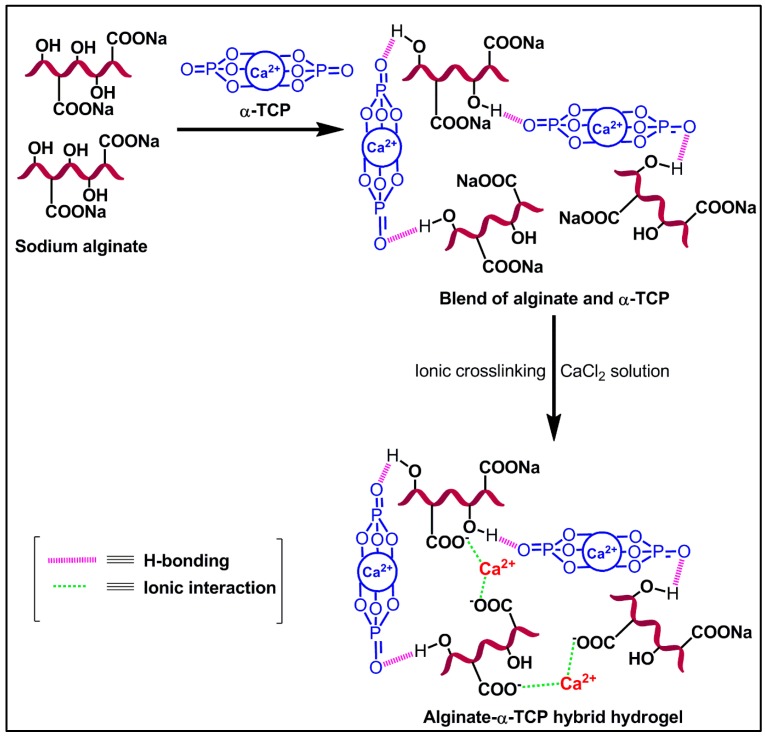
Schematic of probable reaction mechanism for Alg-α-TCP hybrid gel formation.

The ionic cross-linked Alg-α-TCP gel has been synthesized using a slurry of Alg and α-TCP, which has been prepared by ultrasonication and then CaCl_2_ used for crosslinking reaction. The fabrication procedure and mechanism are represented in Figure 1 and Figure 3, respectively. In α-TCP, Ca^2+^ and PO_4_^3−^ form unit cells of α-TCP where, two kinds of columns are present: (i) Ca^2+^ cations columns (C-C) and (ii) Ca^2+^ cations and PO_4_^3−^ anions columns (C-A). Each C-C column is surrounded by six C-A columns [50]. Here, ultra-sonication method has been applied for the homogeneous distribution α-TCP particles in the Alg network and to improve the area of contact for attachment of ceramic particles in the Alg network. In presence of CaCl_2_, the –COO^−^ groups of Alg crosslinked with Ca^2+^ ions through electrostatic interaction and form ionic cross-linked Alg-α-TCP gel (Figure 3). In presence of cross-linker (Ca^2+^), alginate moieties connect with each other through cooperative mechanism and form “egg-box” pattern [49,52]. Basically, the divalent cations (Ca^2+^) connect alginate chains via ionic interaction and make interconnected layers around the eggs box [49,52].

### 3.3. Effect of α-TCP Particles and Ionic Concentration (CaCl_2_) on Gel Formation

**Figure 4:**
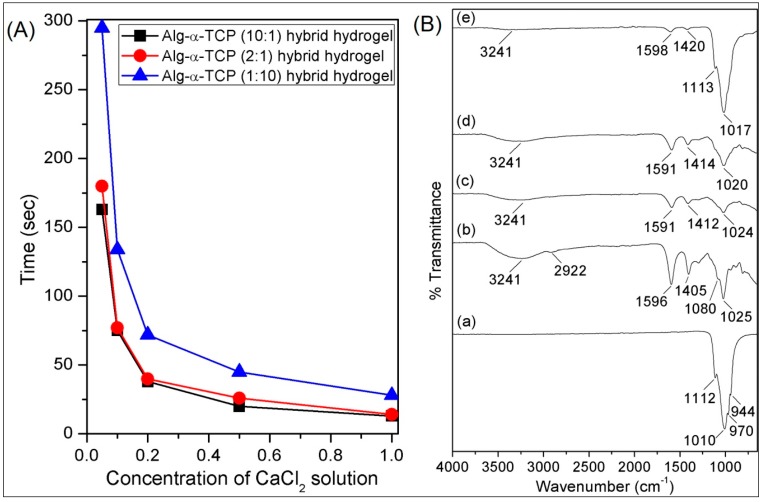
(**A**) Gelation time of Alg-α-TCP (10:1, 2:1 and 1:10) hybrid gels at different concentrations of CaCl_2_ solutions [M] and (**B**) attenuated total reflectance Fourier transform infrared (ATR-FTIR) spectra of dried (**a**) α-TCP, (**b**) sodium alginate and hybrid gel with different compositions (**c**) 10:1, (**d**) 2:1 and (**e**) 1:10.

To observe the effects of composition of the hybrid gel on gel formation, three grades of Alg/α-TCP slurry (10:1, 2:1 and 1:10 *w*/*w*) have been prepared by changing the number of α-TCP particles in 6% Alg solution. Gelation has been visibly observed when the slurry formed round shaped gel masses in the 0.05, 0.1, 0.2, 0.5 and 1.0 M of CaCl_2_ solutions. With progress of time, slurry became hard and formed round shape, which is because of the crosslinking reaction between Alg and α-TCP. After completion of the crosslinking, gel mass detached from the bottom of the Teflon vials. Time has been measured by stop watch. It has been observed that with increase of α-TCP particles gelation time decreased (Figure 4A) in all concentration of CaCl_2_ solutions. For the 10:1 mixture, the gelation time is less than that of 2:1 and 1:10 mixtures. In the 10:1 system, the number of α-TCP particles is less and as a consequence the diffusion of Ca^2+^ ions into the hybrid system is easier compared to those of 2:1 and 1:10 systems. Therefore, Ca^2+^ ions can quickly enter into the system and formed ionic cross-linked network. While in the cases of the 2:1 and 1:10 systems, the presence of higher amount of α-TCP particles create a barrier for the diffusion of Ca^2+^ ions. Consequently, for the diffusion of Ca^2+^ ions into the 2:1 and 10:1 hybrid systems longer time is required than that of 10:1 system. Thus, the gelation time of the Alg: α-TCP hybrid gel follows the order: 10:1 > 2:1 > 1:10. 

From the Figure 4A, it is also noticed that gelation time decreases with increase of ionic concentrations of CaCl_2_. This is due to the fact that the availability of greater numbers of Ca^2+^ ions at higher concentration of CaCl_2_ solution assisted quicker crosslinking process compared to that of lower concentration of CaCl_2_ solution. Hence, the order follows is: 0.05 M < 0.1 M < 0.2 M < 0.5 M < 1.0 M. Here, 0.1 M CaCl_2_ has been selected for the whole experiment.

### 3.4. Characterizations 

In the FTIR spectrum of α-TCP (Figure 4B(a)), the peaks at 1112, 1010, 970 and 944 cm^−1^ are due to the asymmetric and symmetric stretching vibrations of P–O bonds, respectively [42]. In the FTIR spectrum of Alg (Figure 4B(b)), the peaks at 3241, 1596, 1405, 1080 and 1025 cm^−1^ are because of the –OH stretching, stretching of carboxylate groups (C=O, –C–O), asymmetric and symmetric stretching of C–O–C bond, respectively [42]. The FTIR spectra of the dried Alg-α-TCP (1:10, 2:1 and 1:10) hybrid gels are depicts in Figure 4B(c–e). For all hydrogels (Figure 4B(c–e)), it is obvious that the intensities of the stretching vibrations of carboxylate group (1596 and 1405 cm^−1^) decreased which are because of the ionic crosslinking between the carboxylate groups of Alg and Ca^2+^ ions (Figure 3) [42]. Moreover, the progressive broadness of –OH stretching vibration (3241 cm^−1^) indicates that there is a physical interaction (H–bonding) between α-TCP particles and Alg (Figure 4B(c–e)).

**Figure 5:**
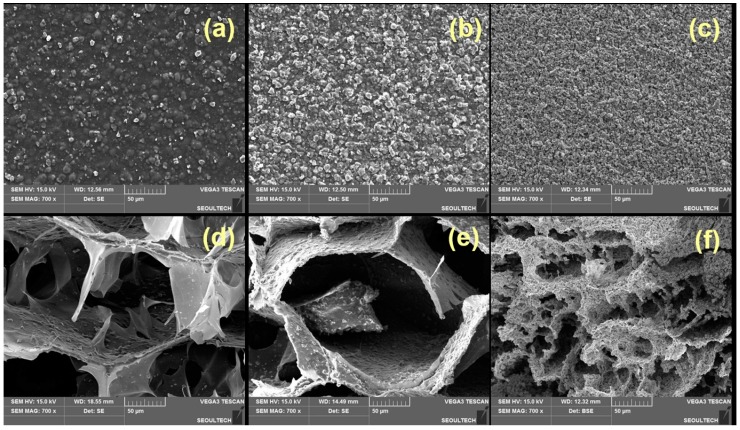
Surface (**a**–**c**) and cross-section images of dried Alg-α-TCP hybrid gels with different compositions (**d**) 10:1, (**e**) 2:1 and (**f**) 1:10.

Figure 5 describes the surface morphology of the dried hybrid gels at different ratios (10:1, 2:1 and 1:10) of Alg and α-TCP. After incorporation of α-TCP particles and ionic crosslinking, the morphology of alginate changed and the hybrid gels appeared with rough surface (Figure 5a–c). It is also noticed that the α-TCP particles are distributed on the Alg surface (Figure 5a–c). In the 1:10 hybrid hydrogel (Figure 5c), α-TCP particles are agglomerated in the surface and formed comparatively roughest surface among all samples. From the cross-section images (Figure 5d–f), it is observed that hydrogels are porous and porosity depends on the compositions. It is also noticed that the α-TCP particles are not only distributed on the surface of the hydrogel but also fully incorporated into the hydrogel network (Figure 5d–f). The regular distribution of α-TCP particles into the Alg network could improve the interfacial interaction between Alg and α-TCP which may increase the mechanical strength of the hybrid gel. The high density of α-TCP particles in 1:10 system form agglomeration and which results smaller pore sizes.

### 3.5. Determination of Calcium Content in Alg-α-TCP Hybrid Gels

The calcium (Ca) content in the Alg-α-TCP hybrid gels have been measured after treating with 70% nitric acid (HNO_3_) solution by inductively coupled plasma mass spectrometry (ICP-MS). It has been observed that the average concentrations of Ca in the nitric acid treated solutions of dried Alg-α-TCP (10:1, 2:1 and 1:10) gels were 12,581,000, 21,924,500 and 44,028,500 ppb (µg/L), respectively.

### 3.6. Swelling Study

**Figure 6:**
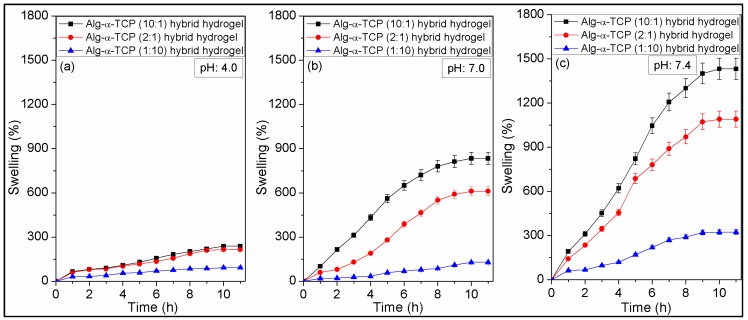
Swelling behaviors of Alg-α-TCP (10:1, 2:1 and 1:10) hybrid gels at (**a**) pH 4.0; (**b**) pH 7.0; (**c**) pH 7.4 and 37 °C.

Figure 6 describes swelling tests results of the Alg-α-TCP (10:1, 2:1 and 1:10) hybrid gels in different pH (4, 7 and 7.4) at 37 °C, where gels attained equilibrium swelling state at ~10 h. It is noticed that % swelling of the 10:1 hybrid gel is higher than those of 2:1 and 1:10 hybrid gels. The phenomena could be explained by the fact that swelling of the hybrid gels depend on the existence and availability of functional hydrophilic groups, crosslinking density and distribution of the filler (α-TCP) into the hybrid gels [49]. For the 10:1 hybrid gel, lower amount of α-TCP is present, while it is highest for the 1:10 hybrid gel. The higher % α-TCP decreased the void space in the gel network and created smaller sizes of pores. This is also noticed in the cross-section images of the hybrid gels (Figure 5d–f). For this reason, the diffusion of water molecules is lowest for 1:10 hybrid gel, compared to those of 2:1 and 10:1 system. Again, from the ATR-FTIR spectra (Figure 4B-b–d), it is observed that the –OH groups are connected by the physical interactions with α-TCP particles. Thus, the availability of hydrophilic –OH groups (which can bind water molecules during swelling) are highest in case of the 10:1 hybrid gel and lowest for the 1:10 hybrid gel. Hence, the % swelling of the hybrid gels followed the order is: 10:1 > 2:1 > 1:10. 

The effect of pH on the swelling behavior of the Alg-α-TCP (10:1, 2:1 and 1:10) hybrid gels is also obvious from Figure 6. It is observed that % swelling is higher at basic medium (pH 7.4) and lower at acidic medium (pH 4). For example, the % swelling of 10:1 hybrid gel was 92 ± 4% at pH 4, 128 ± 6% at pH 7 and 323 ± 16% at pH 7.4. The results are may be because of the fact that at acidic medium (pH 4, Figure 6a), the charge density on hybrid gel decreased as the unreacted carboxylate ions protonated (generally G units form ionic crosslinking with Ca^2+^) [24]. Therefore, the hydrophilicity of the hydrogel decreased at acidic medium, resulting lower % swelling. While at pH 7, no such changes occurred, the crosslinked network remain as integral network. On the other hand, at PBS (pH 7.4), the crosslinking agent (Ca^2+^ ion) takes part in complexation reaction with the ions present in the medium and may form Ca_3_(PO_4_)_2_ or Ca(OH)_2_ as precipitation. In this case, with progress of time, turbidity in the solution has been noticed. Thus, crosslinking density reduced at pH 7.4, which assisted more water absorption. Moreover, at pH 7.4, the unreacted carboxylate ions repulse each other which offers more space for the diffusion of water into the crosslinked network. The weight loss of hybrid gel has been observed at pH 7.4 after 12 h. This is may be because of the breakdown of total ionic crosslinking. Hence, the % swelling follows the order at different pH is: pH 7.4 > pH 7.0 > pH 4.0.

It is reported that α-TCP hydrates after immersion in aqueous media even at 37 °C and forms calcium-deficient hydroxyapatite (CDHAp) [53,54]. The rate of α-TCP hydration also increased with temperature and decreased with increasing pH of the medium [53]. Heat is evolved during the conversion of α-TCP and setting take place [53]. At the initial stage, heat evolution rate is higher and later stage it is lower [53]. The conversion results small crystalline microstructure plate which are interconnected to each other [53]. It is also reported that the orientation of the plate was perpendicular to α-TCP which allows ion transport and do not retard the rate of hydrolysis [53]. The evolution of heat during the transformation process, the particle size and orientation of CDHAp may affect the swelling characteristics of the hydrogel. Due to evolution of heat, the mobility of the water molecule will be increased which may cause faster swelling at the initial stage than later stage. Furthermore, owing to the formation of smaller size CDHAp, the porosity into the hydrogel network will decrease which results slower rate of water diffusion into the hydrogel network in the later stage than that of initial stage.

### 3.7. MC3T3 Cell Responses to the Hybrid Gel

**Figure 7:**
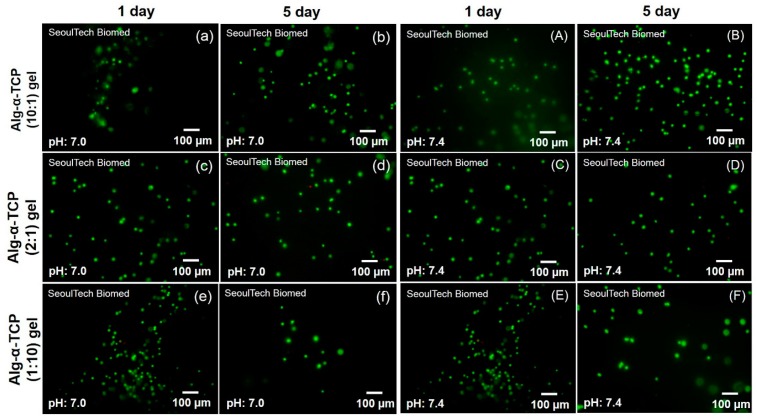
Fluorescence microscopy images of the live and dead MC3T3 cells inside the Alg-α-TCP hybrid gels (1:10, 2:1 and 10:1) at pH 7.0 (**a**–**f**) and pH 7.4 (**A**–**F**) using Calcein AM and EthD-1 kits.

**Figure 8:**
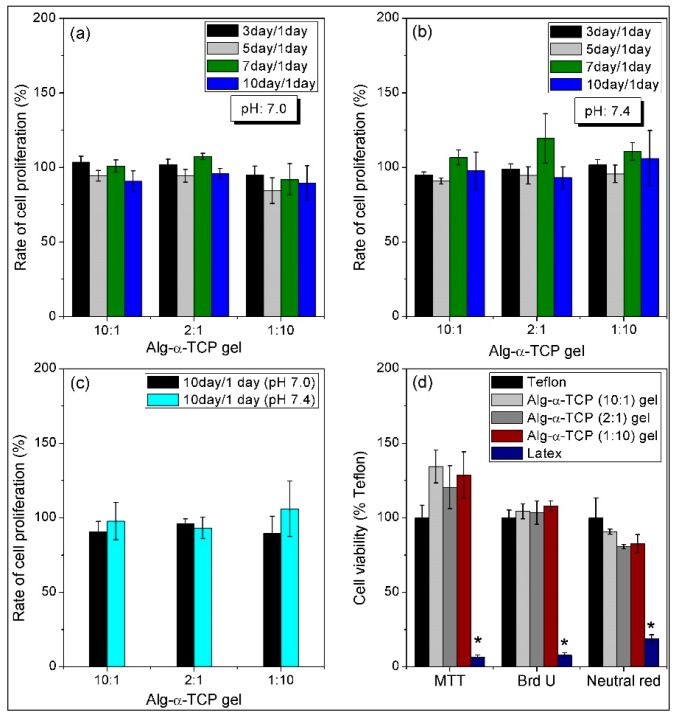
Rate of MC3T3 cell proliferation inside the Alg-α-TCP (10:1, 2:1 and 1:10) gels at (**a**) pH 7.0; (**b**) pH 7.4; (**c**) comparative study of cell proliferation at pH 7.0 and pH 7.4 after 10 days and (**d**) cytotoxicity of hybrid gels by the MTT, BrdU and Neutral red assays.

Figure 7 shows the fluorescence microscopy images of the live and dead MC3T3 cells (passage (*p*) = 12) inside the Alg-α-TCP (10:1, 2:1 and 1:10) hybrid gels at pH 7, 7.4 after 1 and 5 days. It is observed that all the cells were alive inside the hybrid gels after 1 day (Figure 7). However, few numbers of dead cells are obvious in each case after 5 days than that of day 1. The in vitro MC3T3 cell proliferation inside the Alg-α-TCP hybrid gels at different pH was measured up to 10 days by normalizing the cells number with day 1. From the proliferation plots (Figure 8a–c), it is obvious that the rate of cell proliferation increased up to 7 days but at 10 days the rate decreased. This result may be because of the unavailability of space for further cells grow inside the hydrogel. The rate of cell proliferation is higher at pH 7.4 compared to pH 7 (Figure 8c). At basic medium (pH 7.4), the free carboxylate ions of the hybrid gel create repulsive force and stretch the network. Consequently, the space for MC3T3 cells attachment and proliferation is enhanced (Figure 8a–c). The above results indicate that MC3T3 cells are viable inside the Alg-α-TCP hybrid gel network.

### 3.8. Cytotoxicity Study of Hybrid Gel Using MTT, BrdU and Neutral Red Assays

The in vitro biocompatibility of the Alg-α-TCP hybrid gel has been evaluated by measuring the cytotoxicity against specific cell organs (mitochondria, lysosome and DNA) using MTT, Neutral Red and BrdU assays.

Figure 8d represents the cell viability results of the extracts of Teflon, latex and the hybrid gels. The extracts have been prepared after immersing the hybrid gels in media for 3 days and used for the study. Teflon and latex films has been used as positive and negative controls, respectively. The cytotoxicity of the hybrid gels against MC3T3 cells have been measured by comparing the optical densities of both Teflon and latex. The hybrid gels showed better cell viability than that of positive control (Teflon) in both MTT and Brd U assays (Figure 8d). The neutral red assay results showed lower % cell viability in hybrid gels than teflon, however, the viability is more than 90% and no significant difference is observed. Moreover, in all assays, the hybrid gels demonstrated much higher cell viability than negative control (latex). The results confirmed that the Alg-α-TCP hybrid gel is biocompatible and non-toxic.

### 3.9. In Vitro Protein (BSA) and Drugs (TCN and DMOG) Release Study from Alg-α-TCP Hybrid Gel

**Figure 9:**
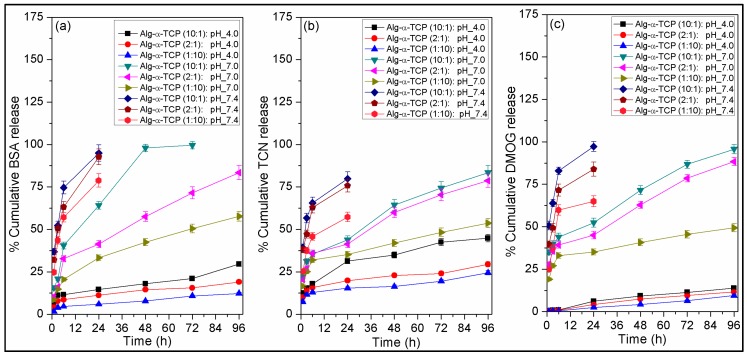
Plots of release of (**a**) BSA; (**b**) TCN and (**c**) DMOG from different grades of Alg-α-TCP (10:1, 2:1 and 1:10) hybrid gels at pH 4.0, 7.0 and 7.4 and 37 °C.

Figure 9 depicts the in vitro release study results of BSA, TCN and DMOG from different grades of Alg-α-TCP (10:1, 2:1 and 1:10) hybrid gels. The release study has been performed at pH 4, 7 and 7.4 and 37 °C.

From the Figure 9a–c, it is observed that at all media, the release rate of BSA, TCN and DMOG is lower for 1:10 hybrid gel, whereas, it is higher for 10:1 hybrid gel. The 10:1 hybrid gel released ~30% BSA in 4 days at pH 4, ~100% BSA in 3 days at pH 7 and ~95% BSA in 1 day at pH 7.4 (Figure 9a). The same hybrid gel released ~45% TCN in 4 days at pH 4, ~84% TCN in 4 days at pH 7 and ~80% TCN in 1 day at pH 7.4 (Figure 9b). It also released ~14% DMOG in 4 days at pH 4, ~96% DMOG in 4 days at pH 7 and ~97% DMOG in 1 day at pH 7.4 (Figure 9c). This can be explained by the fact that for 10:1 hybrid gel, % swelling and swelling rate are higher (Figure 6) because of the presence of lowest amount of α-TCP, greater void space and larger pore sizes. For hydrogel system, rate of swelling is proportional to the drug release rate [55,56]. Consequently, diffusion rate of water molecules into the hybrid gel is higher for 10:1 hybrid gel which resulted higher % protein/drug release. In case of 1:10 hybrid gel, due to the presence of higher amount of α-TCP particles water molecules sense a barrier during the time of diffusion into the crosslinked gel network. As a result, 1:10 hybrid gel showed lowest amount of protein/drug release among three grades of gel. The release order followed is: 10:1 > 2:1 > 1:10. From the Figure 9a–c, it has also been observed that release of BSA, TCN and DMOG significantly depends on the pH of the medium. Their release rates are higher at pH 7.4 and lower at pH 4.0. As described in the swelling section that % swelling and rate of swelling is highest for 10:1 system and lowest for 1:10 system. Thus, the release behaviors of BSA, TCN and DMOG followed the order at different buffers is: pH 7.4 > pH 7.0 > pH 4.0 (Figure 9a–c). On the other hand, it is expected that the heat evolution during the transformation of α-TCP to calcium-deficient hydroxyapatite (CDHAp), may also affect the release rate of drugs. It is assumed that the evolution of heat increased the diffusion of drugs from the hydrogel to media and thus release rate initially increased. Moreover, the extended periods of release of low molecular weight drugs (TCN and DMOG) indicates that drugs molecules formed stronger interaction with the newly developed small sized CDHAp particles and extended the release phenomena. In the literature, it is reported that ~60–80% BSA was released from BSA-loaded alginate microcapsules after 24 h in acidic media [57]. There was a fast BSA release of 70% in 5 h from alginate microspheres at pH 7.4 reported by Cetin et al. [58]. Cetin et al. described that the distraction of the calcium alginate matrix happened faster in a phosphate buffer above pH 5.5 owing to the chelating action of phosphate ions [58]. At pH 7.4, the affinity of calcium to phosphate was higher than that to alginate and subsequently, BSA was released from alginate microspheres through the constant erosion of the microspheres [58]. The erosion of the microspheres could cause the fast release of BSA from the microspheres [58]. Albumin release was dominated by a large initial burst and nearly 100% BSA from the alginate/CaCl_2_ microspheres in about 6 h [59]. Lin et al. reported ~77–95% tetracycline release from alginate fibers at 6 h in PBS [60]. Srisuwan et al. depicted that because of the fast dissolution of the alginate film matrix at pH 7.4, it released ~95% of the loaded tetracycline within 3 h [61]. The above results showed that after modification with α-TCP, the Alg-α-TCP gel released BSA, TCN and DMOG in a controlled way compared to reported native alginate matrix.

### 3.10. Probable Interaction between Protein (BSA), Drugs (TCN and DMOG) and Hybrid Gel

**Figure 10:**
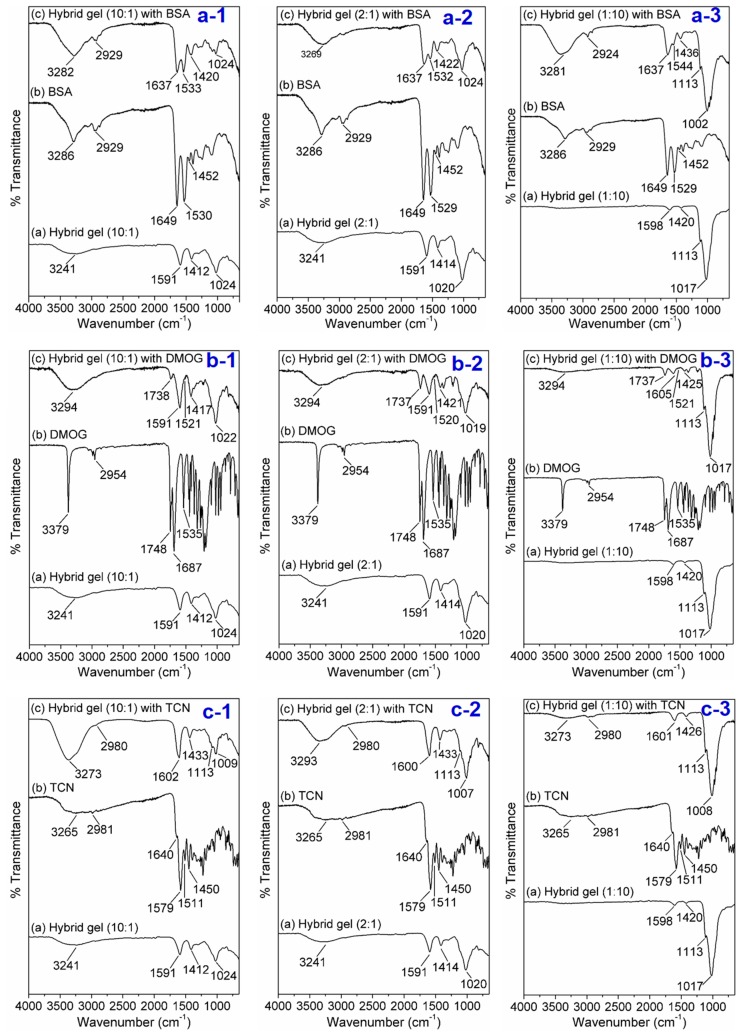
FTIR spectra for the detection of probable interaction between BSA (**a-1–3**), DMOG (**b-1–3**), TCN (**c-1–3**) and Alg-α-TCP (10:1, 2:1 and 1:10) hybrid gels.

The release nature of protein/drugs from the cross-linked gel matrix depends on various factors like pH of the medium, temperature, size of the bioactive molecules, gel strength, swelling rate, % swelling as well as the interaction between bioactive molecules and matrix [55,56]. The molecular weights of BSA, DMOG and TCN are ~66 kDa, 175.1 Da and 444.4 Da, respectively [49]. The comparatively slow release rate of DMOG and TCN suggests that they are strongly bound (stronger interaction) with the hydrogel compared to BSA. The possible interaction between BSA/TCN/DMOG and the hybrid gel has been identified by FTIR analysis. It is apparent that physical interaction, mainly H-bonding is responsible to bind the drugs into the Alg-α-TCP hydrogel. Briefly, from the Figure 10a(1–3), it is apparent that the peaks in the FTIR spectrum of BSA at 3286, 2929, 1649, 1530 and 1452 cm^−1^ are because of the N–H/O–H stretching, C–H stretching, stretching vibrations of amide I, amide-II of peptide unit and stretching vibration of –COO^−^ group of BSA, respectively. From the FTIR spectra of BSA-incorporated hybrid gels it has been noticed that all the characteristics peaks i.e., for N–H stretching, amide-I, amide-II are present in the spectra. However, the peaks for N–H bond at 3286 cm^−1^ broaden and the peaks value for amide-I shift from 1649 to 1637 cm^−1^ which indicate there is a physical interaction between BSA and hybrid gel. In the FTIR spectrum of TCN (Figure 10b(1–3)), the peaks at 3265, 2981, 1640, 1579, 1511, 1450 cm^−1^ are due to N–H/O–H stretching (intramolecular H–bonded), C–H stretching, amide-I, stretching vibrations of –C=O of *keto-enol* functional ring, amide-II stretching and C=C group, respectively. However, the broad peak at 3265 cm^−1^ merged with the peak of –OH group of Alg and appeared at 3293 cm^−1^ in the FTIR spectra of TCN-incorporated hybrid gel. Besides, the shifting of peaks of amide-I and amide-II (1600 and 1426 cm^−1^) indicates physical interaction (H–bonding) and good compatibility between TCN and hybrid gel. From the FTIR spectrum of DMOG (Figure 10c(1–3)), it is apparent that the peaks at 3379, 2954, 1748, 1687 and 1535 cm^−1^ are responsible for the N–H stretching, –C=O of ester group, amide-I and amide-II stretching vibrations, respectively. While in the FTIR spectra of DMOG-incorporated hybrid gels, the broadness and merge of N–H peak (3294 cm^−1^) and shifting of –C=O of ester group (1737 cm^−1^), amide-I and amide-II stretching vibrations (1605, 1591, 1520, 1521 cm^−1^) imply good compatibility between DMOG and hybrid gel, followed by physical interaction. 

## 4. Conclusions

After synthesis of Alg-α-TCP hydrogel by ionic crosslinking, diverse gel responses studies such as gelatin time, morphological changes, swelling, encapsulated cell viability as well as release behavior of bioactive molecules have been carried out. While the physicochemical analyses of the hybrid gel have been performed to verify its structure and components and to understand the mechanisms of swelling characteristics, in vitro drug/protein release behaviors and in vitro cellular responses under different pH. The ATR-FTIR analysis suggested ionic crosslinking happened in presence of Ca^2+^ ions. The SEM analysis showed that the α-TCP are distributed on the surface of the hybrid gel as well as are embedded into the porous crosslinked gel network. The stimuli-responsiveness of the hybrid gel has been ascertained from the pH-sensitive swelling behaviors. The amount of filler (α-TCP particles) greatly affected the swelling characteristics of the hybrid gel, where, higher % α-TCP induced lower % swelling. In vitro MC3T3 cell proliferation and viability inside the hybrid gel confirmed that the synthesized hybrid gel is biocompatible. The pH variation cell study suggested that cell proliferation is higher at pH 7.4 rather than pH 7. The in vitro cytotoxicity analyses using MTT, BrdU and neutral red assays ascertained that the Alg-α-TCP hybrid gel in non-toxic for MC3T3 cells. The hybrid gel released protein (BSA) and drugs (TCN, DMOG) in higher rate at pH 7.4 than those of pH 7.0 and pH 4.0. FTIR spectra ascertained that BSA, DMOG and TCN molecules reside into the Alg-α-TCP matrix by physical interaction especially through H-bonding. The drugs/protein release results showed that the Alg-α-TCP hybrid gel released BSA, DMOG and TCN in controlled way. The experimental results signified that the biocompatible Alg-α-TCP hybrid gel could potentially be employed as scaffold in bone tissue engineering, along with as future ink for 3D bioprinting technology.
